# Association between polyphenol subclasses and prostate cancer: a systematic review and meta-analysis of observational studies

**DOI:** 10.3389/fnut.2024.1428911

**Published:** 2024-07-31

**Authors:** Yiping Huang, Wenyan Wang, Jianxiang Jin

**Affiliations:** ^1^Department of Urology, Affiliated Jinhua Hospital, Zhejiang University School of Medicine, Jinhua, Zhejiang, China; ^2^School of Pharmaceutical Sciences, Key Laboratory of Biotechnology and Pharmaceutical Engineering, Wenzhou Medical University, Wenzhou, Zhejiang, China; ^3^Affiliated Jinhua Hospital, Zhejiang University School of Medicine, Jinhua, Zhejiang, China

**Keywords:** prostate cancer, polyphenol subclasses, phytoestrogen, risk assessment, meta-analysis

## Abstract

**Background:**

The effect of polyphenol subclasses on prostate cancer (PCA) is controversial. Therefore, the purpose of this study was to investigate the relationship between polyphenol subclasses and PCA incidence.

**Methods:**

From the establishment of the database to December 2023, a systematic search was conducted on PubMed, Web of Science, Embase, and Cochrane Library to identify relevant observational studies. The adjusted odds ratio (OR) and corresponding 95% confidence interval (95% CI) were used to assess the association.

**Results:**

A total of 38 studies (11 were cohort studies and 27 were case–control studies), composing 824,933 participants, were included in this meta-analysis after excluding irrelevant records. The findings of the study revealed that men who consumed dietary polyphenols had a significantly higher risk of PCA compared to those who never or rarely consumed dietary polyphenols (OR = 1.01, *p* = 0.023), especially dietary flavonol (OR = 1.05, *p* = 0.042), flavanol (OR = 1.03, *p* = 0.026) and anthocyanin (OR = 1.06, *p* = 0.001). Neither total nor subclasses of dietary polyphenols have an effect on non-localized or high-grade PCA (OR = 1.01, *p* = 0.518). Dietary isoflavones tended to reduce the incidence of local or low-grade PCA, although there was no statistically significant difference (OR = 1.00, *p* = 0.081). Regarding serum/plasma polyphenol, total polyphenol (OR = 0.95, *p* = 0.002), genistein (OR = 0.92, *p* = 0.029) and enterolactone (OR = 0.92, *p* = 0.022) can reduce the incidence of PCA. No association was observed between total/subclasses of urinary polyphenols and PCA risk.

**Conclusion:**

Polyphenols seem to generally increase the risk of PCA in the male population. The effect of polyphenols on PCA is affected by factors such as polyphenol subclasses, their forms (serum/plasma, urinary, dietary), and PCA-related factors (like PCA stage).

**Systematic review registration:**

identifier: CRD42022322699.

## 1 Introduction

Prostate cancer (PCA) is the most frequently diagnosed cancer among men in over half of the world’s countries and was the sixth leading cause of cancer mortality in 2020 (1). Nevertheless, the etiology of PCA has remained poorly understood compared to other common cancers (2). It is widely believed that both endogenous and exogenous factors may influence the occurrence of PCA. Established risk factors for PCA include advancing age (3), race (4), and a family history of PCA (5), all of which are non-modifiable. Numerous modifiable factors, such as smoking (6) and obesity (7), have also been implicated in the development of PCA. Among these factors, nutrition and lifestyle habits, which are more readily changeable, are considered potential avenues for effective cancer prevention strategies (8).

In recent years, Asian diets rich in plant polyphenols and estrogens, such as soybeans, have been associated with a reduced risk of PCA (9, 10). Polyphenols constitute a group of biologically active compounds widely distributed in plants and plant-based foods, including fruits, vegetables, tea leaves, coffee beans, wine, soybeans, lentils, and peanuts (11). They are primarily categorized into four subclasses: flavonoids (including isoflavones and coumestans), phenolic acids, lignans, and stilbenes (12). Among them, isoflavones and lignans have received the most attention in studies. Common isoflavones include daidzein, genistein, glycitein, formononetin, biochanin. Equol, a metabolite of daidzein produced by intestinal bacteria, has also been studied for its biological effects (13). Polyphenols can also be subdivided into many subclasses depending on the number of phenol units within their molecular structure, substituent groups, and the linkage type between phenol units (14).

Experimental studies (15, 16) conducted on cell lines and animal models (17, 18) have demonstrated that polyphenols possess antioxidative and anti-inflammatory effects. They can regulate androgen receptors and/or activate signaling pathway, induce cell cycle arrest and apoptosis, and inhibit the migratory and invasive capabilities of tumor cells. Consequently, they are expected to be used as chemo-preventive drugs for PCA (19). However, the evidence from epidemiological studies is still limited and vague. For example, (i) some studies [e.g., S. S. Strom et al. (20) and Yoshie Nagata et al. (21)] did not observe an association between polyphenol and PCA risk (*p* > 0.05); (ii) subgroup analyses of polyphenol subclasses, PCA stage, etc., showed a high degree of inconsistency across studies (22, 23); (iii) most published observational studies focus only on dietary isoflavones and lignans as polyphenols (24, 25), and the relationship between polyphenol content in serum or urine and PCA has shown inconsistent results. Hence, based on the aforementioned understanding, this study aims to conduct a systematic review and meta-analysis to examine the association between total/subclasses of polyphenols and PCA risk. Meanwhile, this study also hopes to help clinicians and public health personnel provide better prevention reference for the male population from the perspective of dietary habits.

## 2 Experimental

This meta-analysis was conducted according to the Meta-Analysis of Observational Studies in Epidemiology (MOOSE) guidelines (26) and was registered on PROSPERO (CRD42024493149).

### 2.1 Search strategy

From the inception of the database until December 2023, a systematic search was conducted on electronic databases including PubMed, Web of Science, Embase, and the Cochrane Library to identify studies investigating the relationship between polyphenols and PCA risk. The search terms used were: “prostate cancer” and “polyphenol.” The following related terms were selected for the search: (“prostate cancer” OR “prostate neoplasm” OR “PCa” OR “prostatic carcinoma”) AND (“isoflavone” OR “daidzein” OR “genistein” OR “glycitein” OR “biochanin A” OR “formononetin” OR “flavonol” OR “flavanols” OR “flavones” OR “flavanones” OR “anthocyanins” OR “phytoestrogen” OR “polyphenol” OR “lignans” OR “equol” OR “enterolactone” OR “enterodiol” OR “flavonoids”). The reference lists of relevant articles were searched by 2 researchers to avoid omissions. When referring to duplicate literature, the original article was included if the study was published as an abstract and an original article. Also, if a study was continuously updated and reported, only the most recent or comprehensive articles were included. The population, intervention/exposure, comparison, outcome, and setting (PICOS) criteria were used to describe the research question.

### 2.2 Selection criteria

Two researchers independently conducted a search to select studies from the database and reviewed the titles and abstracts of these articles to determine their eligibility for inclusion. When uncertainties arose, the full text was read or looked through for further selection. If necessary, authors are contacted to obtain additional information about their research. In instance of disagreement, discussions were held with a third researcher. When consensus could not be reached, the study was excluded.

Studies were considered for inclusion if they meet each of the following inclusion criteria: (i) the main exposure of the study was polyphenol and the outcome was a risk of PCA; (ii) a correlation between polyphenol and PCA risk was reported; (iii) the study provided usable outcome of polyphenol and PCA risk; (iv) the study is an observational study (cohort study, case–control study and cross-sectional study). At the same time, articles meeting one of the following exclusion criteria would be excluded: (i) the full text cannot be obtained; (ii) articles were not written in English; (iii) the study was conducted on PCA population and used mortality or recovery rate as the outcome; (iv) the study was published in duplicate; (v) the study had no reference value or control group.

### 2.3 Data extraction and quality assessment

The research data were extracted independently by two investigators, with disagreements arbitrated by a third researcher. The following information was extracted using a pre-determined data collection form: (i) authors; (ii) year of publication; (iii) country; (iv) research cohort; (v) study duration; (vi) follow-up period; (vii) number of PCA cases and participants; (viii) measurement tool; (ix) adjusting factors; (x) relevant data.

The quality of cohort and case–control studies was assessed based on the Newcastle-Ottawa Quality Assessment Scale (NOS), which consists of three quality parameters: selection (4 points), comparability (2 points) and outcomes (3 points). A score of 7 or above indicates high quality.

### 2.4 Objectives and endpoints

The primary purpose of this study was to explore the relationship between polyphenols and the incidence of PCA. The secondary purpose was to investigate the relationship between the incidence of PCA and polyphenol subclasses (e.g., isoflavones, daidzein, genistein), different forms of polyphenols and PCA (e.g., serum/plasma polyphenol, urinary polyphenol, dietary polyphenol), and the association between polyphenol and different PCA subtypes (e.g., advanced PCA, localized PCA). The results, after adjusting for relevant confounding factors were uniformly used for the analysis of the data from the included articles.

### 2.5 Statistical analysis

The Stata software version 12 (StataCorp, College Station, Texas, USA) was used to analyze the data. The study used odds ratio (OR) with 95% confidence intervals (95% CI) to evaluate the relationship between polyphenol and the incidence of PCA. The chi-square test was used to determine heterogeneity, with I^2^ values <30% indicating low heterogeneity, I^2^ values between 30 and 60% indicating moderate heterogeneity, and I^2^ values >60% indicating high heterogeneity. Due to the potential heterogeneity among the included studies, a random-effects model was used to improve the credibility of the results. When more than ten studies were included (27, 28), sensitivity analysis and a publication bias test were performed to evaluate the stability and reliability of the results. Publication bias was assessed using Begg’s test, and a two-tailed *P*-test was used to determine statistical significance, with a *p*-value of less than 0.05 considered statistically significant.

## 3 Results

### 3.1 Study selection and quality assessment

**Figure 1:**
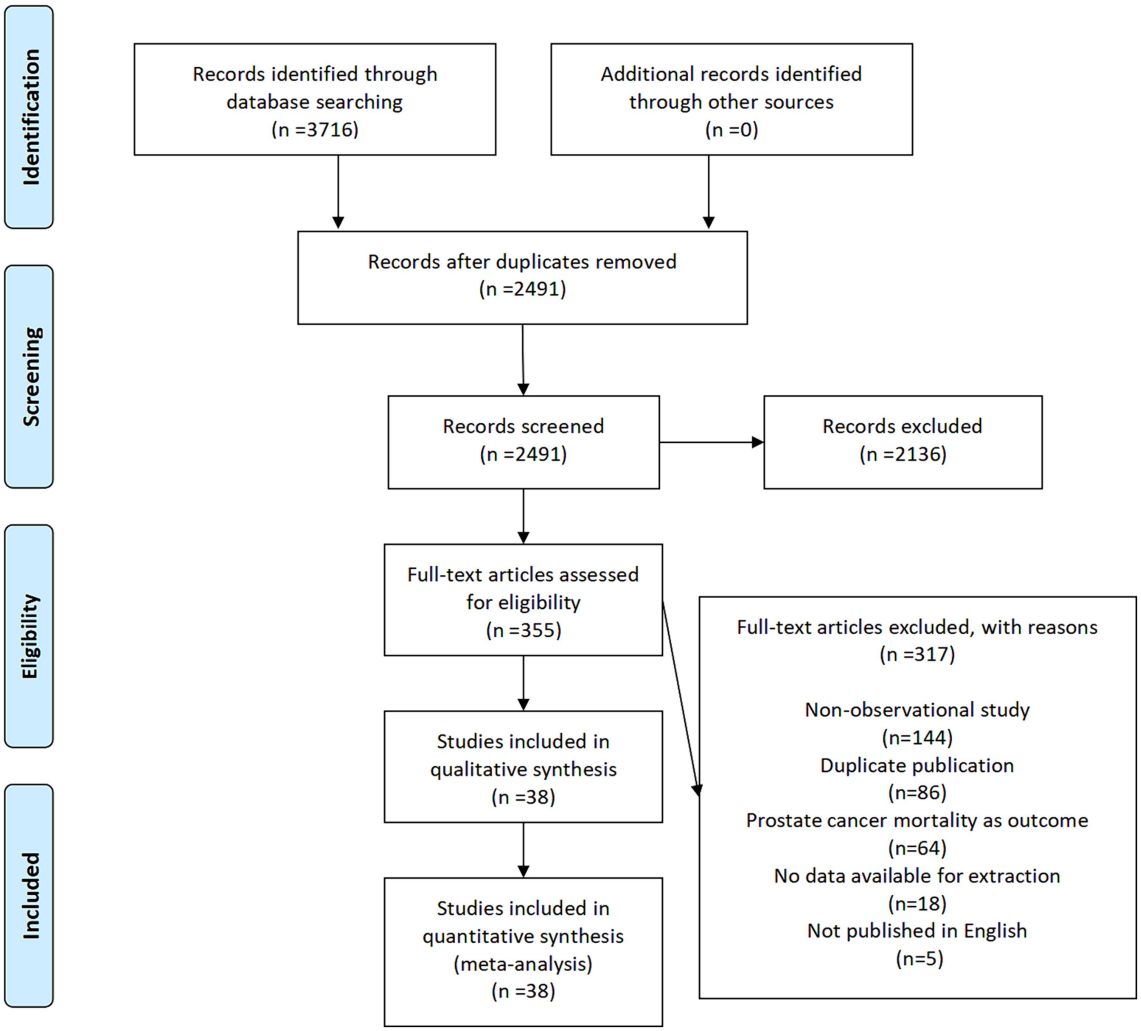
A schematic flow for the selection of articles included in this meta-analysis.

The established retrieval strategy from electronic databases yielded a total of 3,716 records. No additional articles were identified through other sources. After removing duplicates, 2,491 records remained. Subsequently, investigators eliminated 2,136 irrelevant publications after browsing their titles, abstracts, and keywords, leaving 355 articles for full-text review. Among these, 317 articles were eliminated due to reasons such as being non-observational studies (*n* = 144), duplicate publications (*n* = 86), not exploring the risk of PCA (*n* = 64), lack of available data for extraction (*n* = 18), and not being published in English (*n* = 5). As a result of these factors, 38 articles (13, 20–25, 29–59) comprising 824,933 participants met the inclusion criteria. Figure 1 shows the flow diagram of the selection of articles.

Each included observational study in this meta-analysis was assessed across 8 dimensions using the NOS checklist. In cohort studies, 90.9% were of high quality (NOS score ≥ 7), while in case–control studies, 92.6% were deemed high quality (NOS score ≥ 7). The assessment of risk of bias is recorded in Supplementary Tables S2, S3.

### 3.2 Study characteristics

Of the 38 included studies, 11 were cohort studies (795,570 participants and 24,236 PCA cases) and 27 were case–control studies (29,363 controls and 12,888 PCA cases). All studies were published between 1999 and 2023, with follow-up times ranging from 3.5 to 17.3 years. Among them, 15 studies were conducted in Europe, 13 in Asia, and 10 in America. Regarding age at recruitment, 2 studies did not specify an upper age limit, 1 study did not set a lower age limit, and 1 study lacked accessible data. The median age for analysis ranged from 39.3 to 72.5 years, with data lacking in 5 studies. For data collection and evaluation of relevant exposure factors, 31 studies used questionnaires, 15 used blood samples, 4 used interviews, and 2 used urine samples. Additionally, 34.2% of the studies employed a combination of two methods for data evaluation. The adjustment for potential confounding factors varied among the studies, with common parameters including age, geographical area, physical activity, body mass index, family history of PCA, total energy intake, smoking, and alcohol consumption. Tables 1, 2 contain detailed characteristics of the included studies.

**Table 1:** Characteristics of included observational studies in the meta-analysis.

Author, year	Country	Time of experiment (year)	Age at recruitment (year)	Age (median year)	Median follow up time (years)	No. of PCA cases	No. of participants
Knekt P, 2002 (29)	Finland	1966–1972	30–85	39.3	15.6	95	5,218
Greenlee H, 2004 (30)	America	2000–2002	50–76	NA	NA	1,891	35,441
Kurahashi N, 2007 (22)	Japan	1995–2004	45–74	NA	5.0	307	325,371
Mursu J, 2008 (31)	Finnish	1984–1989	42–60	NA	16.2	138	2,590
Park SY, 2008 (32)	America	1993–1996	45–75	NA	8.0	4,404	82,483
Geybels MS, 2013 (24)	Netherland	1986–2003	55–69	62.8	17.3	3,362	58,279
Wang Y, 2014 (23)	America	1999–2009	50–74	70.0	7.8	3,974	43,268
Sawada N, 2017 (25)	Japan	1990–2004	45–74	NA	12.8	307	43,509
Reger MK, 2018 (13)	America	1993–2009	35–70	62.8	11.5	2,598	24,406
Sawada N, 2022 (33)	Japan	1995–2016	45–74	57.6	16.9	221	43,580
Almanza-Aguilera E, 2023 (34)	Europe	1992–2000	35–70	52.2	14.0	6,939	131,425
Strom SS, 1999 (20)	America	1996–1998	35–70	60.8	NA	83	107
Kolonel LN, 2000 (35)	China	1987–1991	<84	70.5	9.5	1,619	1,618
Stattin P, 2002 (36)	Finland, Norway, Sweden	1985–1989	25–64	54.0	9.3	794	2,250
Lee MM, 2003 (37)	China	1989–1992	50–89	60.6	NA	133	265
Ozasa K, 2004 (38)	Japan	1988–1990	>40	69.1	11.0	52	151
McCann SE, 2005 (39)	America	1986–1991	30–75	65.0	NA	433	538
Hedelin M, 2006 (40)	Sweden	2001–2002	35–79	67.2	NA	2,629	1,499
Low YL, 2003 (41)	United Kingdom	1993–1997	45–75	67.6	4.1	89	178
Heald CL, 2007 (42)	Scotland	1998–2001	50–74	66.8	NA	433	483
Bosetti C, 2009 (43)	Italy	1991–2002	46–74	66.0	NA	1,294	1,451
Nagata Y, 2007 (44)	Japan	1996–2003	59–73	60.7	10.2	200	200
Ward H, 2008 (45)	Denmark, Germany, Italy, Spain, Sweden	1993–1997	45–75	66.1	9.5	193	828
Kurahashi N, 2008 (46)	Japan	1990–2005	40–69	58.5	12.8	201	402
Lewis JE, 2009 (47)	America	1998–2004	35–75	63.3	NA	478	382
Travis RC, 2009 (48)	Europe	1992–2000	30–75	60.3	4.2	950	1,042
Park SY, 2009 (49)	America	1993–2006	45–75	69.2	7.3	249	404
Ward HA, 2010 (50)	United Kingdom	1993–2006	40–79	66.1	10.3	203	800
Jackson MD, 2010 (51)	Jamaica	2005–2007	40–80	65.4	5.3	175	194
Sawada N, 2010 (52)	Japan	1990–1993	40–69	58.5	12.5	201	402
Travis RC, 2012 (53)	Europe	1992–2000	43–76	60.1	15.1	1,605	1,697
Sugiyama Y, 2014 (54)	Japan	2011–2013	30–75	64.8	4.0	44	28
Wu Y, 2015 (55)	China	2012–2013	>40	72.5	NA	46	54
Nagata Y, 2016 (21)	Japan	2011–2014	55–73	64.7	5.4	56	56
Russo GI, 2017 (56)	Italy	2015–2016	30–75	69.1	NA	118	222
Reale G, 2018 (57)	Italy	2015–2016	25–75	69.1	NA	118	222
Ghanavati M, 2021 (58)	Iran	2014–2015	40–80	59.8	5.6	97	205
Galván-Portillo M, 2021 (59)	Mexico	2011–2014	25–75	67.7	3.5	395	797

NA, not available; PCA, prostate cancer.

**Table 2:** Characteristics of included observational studies in the meta-analysis.

Author, year	Study type	Polyphenol subclasses	Exposure assessment	Reference value (control group)	Adjusting parameters
Knekt P, 2002 (29)	Cohort study	Flavonoids	Questionnaire	Flavonoids: <4.3 g/day	Sex, age, geographic area, occupation, smoking, body mass index
Greenlee H, 2004 (30)	Cohort study	Isoflavones	Questionnaire	Isoflavones: 0 g/day	Sex, race, education
Kurahashi N, 2007 (22)	Cohort study	Genistein, daidzein	Questionnaire	Genistein: <13.2 mg/day Daidzein: <8.5 mg/day	Age, area, smoking status, drinking frequency, marital status, body mass index, intake of total fatty acids, dairy, vegetables, fruits
Mursu J, 2008 (31)	Cohort study	Flavonols, flavones, flavanones, flavanols, anthocyanidins, flavonoids	Questionnaire	Flavonols: 0 g/day Flavones: 0 g/day Flavanones: 0 g/day Flavanols: 0 g/day Anthocyanidins: 0 g/day Flavonoids: 0 g/day	Age, examination years, body mass index, smoking status, pack-years of smoking, physical activity, intakes of alcohol, total fat, saturated fat, energy adjusted intake of fiber, vitamin C, vitamin E
Park SY, 2008 (32)	Cohort study	Genistein, daidzein, glycitein, isoflavones	Questionnaire	Genistein: <0.7 mg/1,000 kcal Daidzein: <0.7 mg/1,000 kcal Glycitein: <0.18 mg/1,000 kcal Isoflavones: <0.7 mg/1,000 kcal	Time since cohort entry, ethnicity, family history of prostate cancer, education, body mass index, smoking status and energy intake
Geybels MS, 2013 (24)	Cohort study	Flavonols	Questionnaire	Flavonols: 0 g/day	Age
Wang Y, 2014 (23)	Cohort study	Flavonoids, anthocyanidins, flavanols, flavanones, flavones, flavonols, isoflavones	Questionnaire	Flavonoids: <126.6 mg/day Anthocyanidins: <5.9 mg/day Flavanols: <10.4 mg/day Flavanones: <7.5 mg/day Flavones: <0.5 mg/day flavonols: <8.9 mg/day isoflavones: <0.029 mg/day	Age, race, family history of prostate cancer, body mass index in 1999, smoking status, aspirin use, total energy intake, history of prostate-specific antigen screening, history of diabetes.
Sawada N, 2017 (25)	Cohort study	Genistein, daidzein	Questionnaire	Genistein: 0 g/day Daidzein: 0 g/day	NA
Reger MK, 2018 (13)	Cohort study	Isoflavones, genistein, daidzein, glycitein, formononetin, biochanin a, coumestrol	Questionnaire	Isoflavones: <0.17 mg/day Genistein: <0.04 mg/day Daidzein: <0.11 mg/day Glycitein: <0.001 mg/day Formononetin: <0.005 mg/day Biochanin a: <0.028 mg/day Coumestrol: <0.03 mg/day	Age, race/ethnicity, body mass index, smoking status, alcohol intake, family history of prostate cancer.
Sawada N, 2022 (33)	Cohort study	Isoflavones	Questionnaire	Isoflavones: <13.5 mg/day	Age, area, smoking, alcohol frequency, body mass index, leisure time activity, history of diabetes, screening, intake of green tea, coffee, vegetables and fruit
Almanza-Aguilera E, 2023 (34)	Cohort study	Polyphenols, flavonoids, flavanols, flavanols, flavonols, flavanones, anthocyanins, flavones, isoflavones, lignan	Questionnaire	Polyphenols: <783 mg/day Flavonoids: 0 mg/day Flavanols: 0 mg/day Flavanols: 0 mg/day Flavonols: 0 mg/day Flavanones: 0 mg/day Anthocyanins: 0 mg/day Flavones: 0 mg/day Isoflavones: 0 mg/day Lignan: 0 mg/day	Smoking status, physical activity, educational level, marital status, and diabetes prevalence, and alcohol, body mass index, total energy, fiber, vitamin C intakes
Strom SS, 1999 (20)	Case–control study	Isoflavones, genistein, daidzein, formononetin, biochanin a, coumestrol, phytosterols	Questionnaire	Isoflavones: 0 mg/day Genistein: 0 mg/day Daidzein: 0 mg/day Formononetin: 0 mg/day Biochanin a: 0 mg/day Coumestrol: 0 mg/day Phytosterols: 0 mg/day	Age, family history of prostate cancer, alcohol intake, total calorie intake
Kolonel LN, 2000 (35)	Case–control study	Isoflavones	Questionnaire, interview	Isoflavones: 0 g/day	Age, education, ethnicity, geographic area, calories
Stattin P, 2002 (36)	Case–control study	Enterolactone	Blood sample	Enterolactone: <4.32 nmoL/L	NA
Lee MM, 2003 (37)	Case–control study	Genistein, daidzein	Interview	Genistein: <17.9 mg/day Daidzein: <10.0 mg/day	Total calories, age
Ozasa K, 2004 (38)	Case–control study	Genistein, daidzein, equol	Questionnaire, blood sample	Genistein: <239 nmoL/L Daidzein: <89 nmoL/L equol: <1.91 nmoL/L	Age
McCann SE, 2005 (39)	Case–control study	Lignans	Questionnaire	lignans: ≤2 g/day	Age, education, body mass index, cigarette smoking status, total energy
Hedelin M, 2006 (40)	Case–control study	Isoflavones, genistein, daidzein, lignans	Questionnaire, blood sample	Isoflavones: <1.0 g/day Genistein: <0.27 g/day Daidzein: <0.49 g/day Lignans: <17.9 g/day	Age, intake of antibiotics, zinc, animal fat, total energy intake, alcohol, vegetable, fat, carbohydrates, red meat
Low YL, 2003 (41)	Case–control study	Daidzein, genistein, glycitein, equol, enterodiol, enterolactone	Questionnaire, blood sample	Daidzein: 0 μg/mmol creatinine Genistein: 0 μg/mmol creatinine Glycitein: 0 μg/mmol creatinine Equol: 0 μg/mmol creatinine Enterodiol: 0 μg/mmol creatinine Enterolactone: 0 μg/mmol creatinine	Family history of prostate cancer, weight, height, and energy intake
Heald CL, 2007 (42)	Case–control study	Isoflavones, daidzein, genistein, equol, enterolactone	Questionnaire, blood sample	Isoflavones: <58.1 mg/day equol: 0 nmoL/L Daidzein: <8.26 nmoL/L Genistein: <14.23 nmoL/L Enterolactone: <8.14 nmoL/L	Age, total energy intake, family history of prostate cancer and breast cancer, Carstairs Deprivation Index, smoking, energy intake
Bosetti C, 2009 (43)	Case–control study	Flavanones, flavanols, flavonols, flavones, anthocyanidins, isoflavones, flavonoids	Questionnaire	Flavanones: <5.2 mg/day Flavanols: <29.9 mg/day Flavonols: <15.1 mg/day Flavones: <0.2 mg/day Anthocyanidins: <8.3 mg/day Isoflavones: <14.7 mg/day Flavonoids: <109.4 mg/day	Age, study center, education, body mass index, family history of prostate cancer, total calorie intake
Nagata Y, 2007 (44)	Case–control study	Isoflavones, daidzein, genistein	Questionnaire	Isoflavones: <30.5 mg/day Daidzein: <1.1 mg/day Genistein: <0.8 mg/day	Cigarette smoking, energy, fatty acids intakes
Ward H, 2008 (45)	Case–control study	Lignans, isoflavones, genistein, daidzein, equol, glycitein, enterodiol, enterolactone	Questionnaire, blood sample	Lignans: 0 ng/mL Isoflavones: 0 ng/mL Genistein: 0 ng/mL Daidzein: 0 ng/mL Equol: 0 ng/mL Glycitein: 0 ng/mL Enterodiol: 0 ng/mL Enterolactone: 0 ng/mL	Age, height, weight, intake of energy, fat, lycopene, whether sample had been analyzed in a prior publication
Kurahashi N, 2008 (46)	Case–control study	Genistein, daidzein, glycitein, equol	Questionnaire, blood sample	Genistein: <57 ng/mL Daidzein: <22 ng/mL Glycitein: <1.0 ng/mL Equol: <1.0 ng/mL	Smoking status, alcohol intake, marital status, intake of green tea, protein, fiber, green or yellow vegetables
Lewis JE, 2009 (47)	Case–control study	Genistein, daidzein	Questionnaire	Genistein: ≤196 μg/day Daidzein: ≤77 μg/day	Age, education, body mass index, smoking history, family history of prostate cancer in first-degree relatives, total caloric intake
Travis RC, 2009 (48)	Case–control study	Genistein, daidzein, equol, lignans, enterolactone, enterodiol	Blood sample	Genistein: <0.30 ng/mL Daidzein: <0.30 ng/mL Equol: <0.05 ng/mL Lignans: 0 ng/mL Enterolactone: <1.40 ng/mL Enterodiol: <0.10 ng/mL	Smoking, physical activity, alcohol intake, marital status, education, body mass index
Park SY, 2009 (49)	Case–control study	Daidzein, genistein, equol, enterolactone	Urine sample	Daidzein: <0.053 nmol/mg Genistein: <0.009 nmol/mg Equol: <0.0001 nmol/mg Enterolactone: <0.227 nmol/mg	Age at specimen collection, fasting hours, family history of prostate cancer, body mass index, education
Ward HA, 2010 (50)	Case–control study	Phytoestrogens, isoflavones, genistein, daidzein, glycitein, biochanin a, formononetin, lignans, enterolactone, equol, coumestrol	Questionnaire, blood sample	Phytoestrogens: 0 μg/mmol creatinin Isoflavones: 0 μg/mmol creatinin Genistein: 0 μg/mmol creatinin Daidzein: 0 μg/mmol creatinin glycitein: 0 μg/mmol creatinin Biochanin a: 0 μg/mmol creatinin Formononetin: 0 μg/mmol creatinin Lignans: 0 μg/mmol Creatinin enterolactone: 0 μg/mmol creatinin Equol: 0 μg/mmol creatinin Coumestrol: 0 μg/mmol creatinin	Age, height, weight, physical activity, social class, family history of prostate cancer, daily intake of energy, fat, zinc, selenium, lycopene, total intake of dairy products
Jackson MD, 2010 (51)	Case–control study	Genistein, daidzein, equol, enterolactone	Questionnaire, blood sample, urine sample	Genistein: <0.155 nmol/mg creatinine Daidzein: <0.117 nmol/mg creatinine Equol: <0.035 nmol/mg creatinine Enterolactone: <0.550 nmol/mg creatinine	Age, alcohol, body mass index, education, family history of prostate cancer, physical activity, antibiotic use, smoking
Sawada N, 2010 (52)	Case–control study	Genistein, equal	Questionnaire, blood sample	Genistein: <86.2 ng/mL equal: <1.0 ng/mL	Testosterone, sex hormone-binding globulin, smoking status, alcohol intake, marital status, body mass index, intake of green tea and miso soup
Travis RC, 2012 (53)	Case–control study	Genistein	Blood sample	Genistein: <0.30 ng/mL	Smoking, physical activity, alcohol intake, marital status, education, body mass index
Sugiyama Y, 2014 (54)	Case–control study	Genistein, daidzein, glycitein, equol	Blood sample	Genistein: ≤59.3 ng/mL Daidzein: ≤19.3 ng/mL Glycitein: ≤1.0 ng/mL equol: <0.5 ng/mL	Age
Wu Y, 2015 (55)	Case–control study	Genistein	Blood sample, interview	Genistein: <640.2 nmoL/L	Age
Nagata Y, 2016 (21)	Case–control study	Genistein, daidzein, glycitein, equol	Questionnaire, blood sample, fecal sample	Genistein: <57.10 ng/mL Daidzein: <18.00 ng/mL Glycitein: <0.80 ng/mL Equol: <0.50 ng/mL	Age, body mass index, total energy intake, smoking, alcohol status
Russo GI, 2017 (56)	Case–control study	Lignans, isoflavones, daidzein, genistein, glycitein, biochanin a	Questionnaires	Lignans: 0 g/day Isoflavones: 0 g/day Daidzein: 0 g/day Genistein: 0 g/day Glycitein: 0 g/day Biochanin a: 0 g/day	Age, energy intake, weight status, smoking status, Alcohol consumption, physical activity level, family history of prostate cancer
Reale G, 2018 (57)	Case–control study	Flavonoids, anthocyanins, flavonols, flavanols, flavanones, flavones	Questionnaire	Flavonoids: 0 g/day Anthocyanins: 0 g/day Flavonols: 0 g/day Flavanols: 0 g/day Flavanones: 0 g/day Flavones: 0 g/day	Age, energy intake, weight status, smoking status, alcohol consumption, physical activity level, family history of prostate cancer
Ghanavati M, 2021 (58)	Case–control study	Flavonoids, lignans, polyphenols, anthocyanins, flavonols, flavanols, flavanones, flavones	Questionnaire	Flavonoids: <718.29 mg/day lignans: <8.76 mg/day Polyphenols: <2287.19 mg/day Anthocyanins: <23.17 mg/day Flavonols: <438.74 mg/day Flavanols: <94.27 mg/day Flavanones: <58.33 mg/day Flavones: <3.35 mg/day	Energy intake, hypertension, diabetes, smoking, body mass index and waist circumstance
Galván-Portillo M, 2021 (59)	Case–control study	Flavones, flavonols, flavanols	Questionnaire, interview	Flavones: 1.0 mg/day Flavonols: 1.0 mg/day Flavanols: 1.0 mg/day	Age, educational level, history of chronic disease, history of sexually transmitted disease, history of prostate cancer in first-degree relatives, leisure physical activity and smoking patterns throughout life, raw tomato, green-yellow leafy vegetables, green-yellow nonleafy vegetables

### 3.3 Dietary polyphenol and PCA

Twenty-seven studies recorded data on the risk of PCA associated with total intake of dietary polyphenols. The analysis indicated that men who consumed dietary polyphenols had a significantly higher risk of PCA than those who never or rarely consumed dietary polyphenol (OR = 1.01, *p* = 0.023) with moderate heterogeneity (I^2^ = 41.0%). In other words, dietary polyphenol intake was found to be harmful for the male population, increasing PCA risk. Additionally, cohort studies (OR = 1.01, *p* < 0.001) and case–control studies (OR = 1.01, *p* = 0.089) have consistently concluded that the consumption of dietary polyphenol increases the risk of PCA in men. The detailed data are contained in Table 3.

**Table 3:** Effects of dietary polyphenol subclasses on PCA incidence.

Subgroup analysis	No. of studies	OR	95%CI	*p*	Heterogeneity (I^2^) (%)
Nonlocalized or high-grade PCA					
Total polyphenol	27	1.01	1.00–1.02	*0.023*	41.0
Cohort study	9	1.01	1.01–1.02	<0.001***	15.4
Case–control study	18	1.01	1.00–1.02	0.089	52.6
Subclasses of polyphenol					
Isoflavone	14	1.00	0.98–1.03	0.818	27.4
Genistein	14	0.98	0.95–1.00	0.107	13.8
Daidzein	13	1.00	0.95–1.04	0.822	36.7
Glycitein	5	0.98	0.94–1.01	0.229	28.9
Biochanin A	4	0.98	0.93–1.05	0.592	0
Formononetin	3	0.99	0.93–1.05	0.641	0
Coumestrol	4	0.97	0.91–1.02	0.223	9.7
Flavonoid	6	1.02	1.00–1.04	0.082	0
Flavonol	7	1.05	1.00–1.10	0.042*	53.5
Flavanol	7	1.03	1.00–1.05	0.026*	34.1
Flavone	7	1.02	0.97–1.06	0.456	48.3
Flavanone	6	1.01	0.97–1.06	0.583	65.8
Anthocyanin	6	1.06	1.02–1.09	0.001**	31.1
Phytoestrogen	4	0.91	0.81–1.01	0.084	4.4
Lignan	6	0.96	0.88–1.05	0.364	71.9
Other polyphenol	2	1.02	0.95–1.09	0.626	66.2

PCA, prostate cancer; OR, odd ratio; CI, confidence interval. *p < 0.05; **p < 0.01; ***p < 0.001.

### 3.4 Dietary polyphenol subclasses and PCA

Different dietary polyphenol subclasses exhibit varying effects on PCA. The results indicated that dietary flavonol intake (OR = 1.05, *p* = 0.042), dietary flavanol intake (OR = 1.03, *p* = 0.026), and dietary anthocyanin intake (OR = 1.06, *p* = 0.001) were associated with an increased incidence of PCA, with statistically significant differences observed. However, dietary isoflavone (OR = 1.00, *p* = 0.818), genistein (OR = 0.98, *p* = 0.107), daidzein (OR = 1.00, *p* = 0.822), glycitein (OR = 0.98, *p* = 0.229), biochanin A (OR = 0.98, *p* = 0.592), formononetin (OR = 0.99, *p* = 0.641), coumestrol (OR = 0.97, *p* = 0.223), flavonoids (OR = 1.02, *p* = 0.082), flavone (OR = 1.02, *p* = 0.456), flavanone (OR = 1.01, *p* = 0.583), phytoestrogen (OR = 0.91, *p* = 0.084), lignan (OR = 0.96, *p* = 0.364) and other polyphenols (OR = 1.02, *p* = 0.626) did not show a significant effect on PCA, that was, these were not an observable risk factor for PCA in men. The detailed data are contained in Table 3.

### 3.5 Dietary polyphenol subclasses and different PCA stage

For non-localized or high-grade PCA, data from 8 studies on the intake of dietary polyphenol subclasses and PCA risk were available. The analysis revealed that total dietary polyphenol intake had no effect on the occurrence of PCA (OR = 1.01, *p* = 0.518). Additionally, intake of all dietary polyphenol subclasses showed no significant relationship with PCA risk, including genistein (OR = 0.97, *p* = 0.565), daidzein (OR = 0.94, *p* = 0.287), glycitein (OR = 0.99, *p* = 0.867), isoflavone (OR = 1.07, *p* = 0.233), flavonoids (OR = 1.00, *p* = 0.861), flavonols (OR = 1.01, *p* = 0.728), flavanol (OR = 0.99, *p* = 0.618), flavones (OR = 1.01, *p* = 0.847), flavanones (OR = 1.01, *p* = 0.538), and anthocyanidins (OR = 1.01, *p* = 0.474). The detailed data are contained in Table 4.

**Table 4:** Effects of dietary polyphenol subclasses on different PCA stage incidence.

Subgroup analysis	No. of studies	OR	95%CI	*p*	Heterogeneity (I^2^) (%)
Nonlocalized or high-grade PCA					
Total polyphenol	8	1.01	0.99–1.02	0.518	26.6
Subclasses of polyphenol					
Genistein	4	0.97	0.88–1.07	0.565	33.5
Daidzein	4	0.94	0.84–1.05	0.287	41.4
Glycitein	2	0.99	0.86–1.14	0.867	58.0
Isoflavone	3	1.07	0.96–1.20	0.233	54.8
Flavonoid	2	1.00	0.96–1.06	0.861	0
Flavonol	3	1.01	0.97–1.05	0.728	0
Flavanol	2	0.99	0.97–1.02	0.618	0
Flavone	2	1.01	0.95–1.06	0.847	0
Flavanone	2	1.01	0.97–1.06	0.538	8.7
Anthocyanidin	2	1.01	0.98–1.05	0.474	0.9
Localized or low-grade PCA					
Total polyphenol	8	1.00	0.99–1.00	0.081	0.0
Subclasses of polyphenol					
Genistein	4	0.96	0.91–1.01	0.083	28.1
Daidzein	4	0.99	0.95–1.03	0.586	0
Glycitein	2	0.98	0.94–1.02	0.253	0
Isoflavone	4	0.99	0.98–1.00	0.020*	0
Flavonoid	2	1.01	0.98–1.04	0.565	0
Flavonol	3	1.02	0.99–1.06	0.143	4.9
Flavanol	2	1.00	0.98–1.02	0.986	0
Flavone	2	1.02	0.98–1.05	0.306	0
Flavanone	2	0.99	0.97–1.00	0.109	0
Anthocyanidin	2	1.01	1.00–1.03	0.129	0

PCA, prostate cancer; OR, odd ratio; CI, confidence interval. *p < 0.05.

For localized or low-grade PCA, data from 8 studies on the intake of dietary polyphenol subclasses and PCA risk were analyzed. The analysis showed that total dietary polyphenol had no effect on the occurrence of PCA (OR = 1.00, *p* = 0.081). Subgroup analysis revealed that dietary intake of genistein (OR = 0.96, *p* = 0.083), daidzein (OR = 0.99, *p* = 0.586), glycitein (OR = 0.98, *p* = 0.253), flavonoids (OR = 1.01, *p* = 0.565), flavonols (OR = 1.02, *p* = 0.143), flavanols (OR = 1.00, *p* = 0.986), flavones (OR = 1.02, *p* = 0.306), flavanones (OR = 0.99, *p* = 0.109), and anthocyanidins (OR = 1.01, *p* = 0.129) have no effect on PCA incidence, with slight heterogeneity. However, dietary isoflavone intake was found to reduce the incidence of PCA (OR = 0.99, *p* = 0.020), with statistically significant differences. The detailed data are contained in Table 4.

### 3.6 Serum/plasma polyphenol subclasses and PCA

Fourteen studies investigated the effect of serum/plasma polyphenols on PCA risk and twelve studies provided data on serum/plasma polyphenol subclasses. The results indicate that total serum/plasma polyphenol can reduce the incidence of PCA in the male population (OR = 0.95, *p* = 0.002). Among them, serum/plasma genistein (OR = 0.92, *p* = 0.029) and enterolactone (OR = 0.92, *p* = 0.022) showed statistically significant protective effects against PCA, while serum/plasma daidzein (OR = 0.91, *p* = 0.053), glycitein (OR = 0.87, *p* = 0.095), equol (OR = 1.02, *p* = 0.733), and isoflavone (OR = 1.01, *p* = 0.708) has no significant effect on PCA. The detailed data are presented in Table 5.

**Table 5:** Effects of serum/plasma polyphenol subclasses on PCA incidence.

Subgroup analysis	No. of studies	OR	95%CI	*p*	Heterogeneity (I^2^) (%)
Total polyphenol	14	0.95	0.92–0.98	0.002**	50.3
Subclasses of polyphenol					
Genistein	12	0.92	0.85–0.99	0.029*	42.3
Daidzein	9	0.91	0.83–1.00	0.053	50.8
Glycitein	6	0.87	0.74–1.02	0.095	73.3
Equol	10	1.02	0.93–1.11	0.733	40.5
Isoflavone	3	1.01	0.95–1.07	0.708	0
Enterolactone	7	0.92	0.85–0.99	0.022*	61.2

PCA, prostate cancer; OR, odd ratio; CI, confidence interval. *p < 0.05; **p < 0.01.

### 3.7 Urinary polyphenol subclasses and PCA

Four studies explored the effect of urinary polyphenol on PCA risk, and detailed data on urinary polyphenol subclasses were available from four studies. The results showed that total urinary polyphenol had no significant influence on PCA risk (OR = 0.98, *p* = 0.184). Meanwhile, none of the urinary polyphenol subclasses demonstrated a significant effect on the occurrence of PCA in the male population, including genistein (OR = 0.97, *p* = 0.253), daidzein (OR = 0.99, *p* = 0.682), glycitein (OR = 1.00, *p* = 0.985), isoflavone (OR = 0.97, *p* = 0.523), enterolactone (OR = 0.97, *p* = 0.291), and equol (OR = 0.90, *p* = 0.406). The detailed data are contained in Table 6.

**Table 6:** Effects of urinary polyphenol subclasses on PCA incidence.

Subgroup analysis	No. of studies	OR	95%CI	*p*	Heterogeneity (I^2^) (%)
Total polyphenol	4	0.98	0.95–1.01	0.184	17.5
Subclasses of polyphenol					
Genistein	4	0.97	0.92–1.02	0.253	0
Daidzein	4	0.99	0.94–1.04	0.682	0
Glycitein	3	1.00	0.89–1.12	0.985	18.3
Isoflavone	2	0.97	0.89–1.06	0.523	0
Enterolactone	4	0.97	0.92–1.03	0.291	14.2
Equol	4	0.90	0.70–1.15	0.406	71.9

PCA, prostate cancer; OR, odd ratio; CI, confidence interval.

### 3.8 Publication bias and sensitivity analysis

Begg’s test was used to estimate publication bias. The results of Begg’s test indicated the absence of publication bias among the included articles (*p* > 0.05). Sensitivity analysis was performed to assess whether individual studies affected the overall results. The results indicated that the analysis was relatively stable.

## 4 Discussion

This study found that men who consumed dietary polyphenols had a significantly higher risk of PCA compared to those who never or rarely consumed dietary polyphenols, especially dietary flavonols, flavanols and anthocyanins. While total polyphenols and their subclasses did not show an effect on non-localized or high-grade PCA, dietary isoflavones were found to reduce the incidence of localized or low-grade PCA. For serum/plasma polyphenols, total polyphenol intake, as well as genistein and enterolactone, were associated with a reduced incidence of PCA. No significant association was observed between total or subclasses of urinary polyphenols and PCA risk.

Due to the diverse array of polyphenol types (60), there is no unified mechanism to explain the impact of polyphenol on the incidence of PCA. Researchers generally believe that polyphenols found in blood and urine originate from dietary intake, which mainly includes isoflavones, genistein, daidzein, glycitein, formononetin, and biochanin A, which are present in soy, soy-based foods and legumes (61, 62). The biological mechanisms underlying the effects of polyphenol on the human body have been proposed in the past decade. The prevailing perspective suggests that polyphenols exert their potential health effects due to their structural similarity to estrogen (63), which allows them to bind to estrogen receptors and produce both anti-estrogenic and estrogenic effects (55). Additionally, polyphenols can also induce significant cellular changes, particularly through the RAS-MAPK signaling pathways, which may promote the proliferation and metastasis of PCA cells (26). Consequently, polyphenols play various roles in gene regulation, cancer biology, and treatment (64).

According to previous studies, androgen levels significantly influence prostate health, and long-term exposure to androgens or increased cellular response to androgens is a key risk factor for PCA (65). Exogenous polyphenol intake can reduce testosterone production in the testes through a negative feedback mechanism, thereby exerting anti-androgenic effects (66). On the one hand, plant estrogens found in polyphenols can bind to a large number of estrogen receptors in prostate tissue, exerting an antagonistic effect on androgens, thereby regulating tumor cell proliferation and apoptosis, inhibiting angiogenesis and tumor metastasis (67, 68). On the other hand, the intake of flavonoids, a type of polyphenol that is not classified as phytoestrogens, may lead to disruptions in the human endocrine system and induce cellular stress (65, 69). Additionally, exogenous supplementation of phytoestrogens can alter hormone metabolism, potentially converting these hormones into androgens, which may lead to an increase in circulating androgens and reactive oxygen species, increasing the risk of the prostate cancer (70).

Researchers have not reached a unified conclusion on the balance and interaction between these two effects. However, the interplay of genetics, gene–environment interactions (such as race), and specific nutrient exposures can provoke diverse cellular effects, influencing PCA susceptibility (27, 71, 72). The varying degrees of impact observed across different studies can be attributed to background factors that influence the susceptibility of PCA through population-specific gene–environment interactions (73, 74). This may explain why our study found that consuming dietary polyphenols, flavanols, and anthocyanins increases the risk of PCA, while other studies (57, 75) have reported that dietary polyphenols may reduce the risk of PCA. Additionally, research indicates that as PCA progresses, mutations or epigenetic silencing of DNA repair genes in cancer cells can affect their sensitivity to hormone therapy, potentially leading to the progression of tumors from hormone-dependent to hormone-independent (76). This is a crucial issue in the management of advanced-stage cancer (77). This may explain why dietary polyphenol intake has a protective effect on localized or low-grade PCA, while its effect on non-localized or high-grade PCA is not significant.

Most serum/plasma and urinary polyphenols are metabolites of dietary polyphenols produced by gut microbiota, mainly including equol and enterolactone (78). The impact of these polyphenols on the human body is largely related to the polymorphisms associated with individual differences in gut microbiota and their effects on the formation of active metabolites (78, 79). Previous studies have suggested that the polyphenolic metabolite equol, being an estrogenic compound, may be more active than daidzein (80). However, due to individual differences, daidzein is only metabolized to form equol in a portion of the population. Similarly, lignans, a type of polyphenol, can only be converted into enterolactone and enterodiol by the gut microbiota in a portion of the population (63, 81). Different types of polyphenols exhibit distinct absorption and metabolic characteristics in the body. For example, flavonoid polyphenols may have higher bioavailability compared to phenolic acid polyphenols (14, 27). These differences in chemical composition might lead to variations between dietary polyphenols and serum/plasma polyphenol levels. Individual dietary habits, lifestyle choices, and health status also influence polyphenol absorption and metabolism. Factors such as smoking, alcohol consumption, exercise, and medication use can impact polyphenol metabolism and serum/plasma levels (23, 28, 29). Therefore, owing to the complexity of the human diet and the different confounding factors controlled for in various studies, we should be cautious about the conclusions drawn on the impact of polyphenol subclasses on PCA, as any bias may cause changes in the results. Further research is needed to explore how polyphenolic compounds interact with specific genes and how these interactions influence cancer risk and progression, particularly considering the potential differences in their effects among diverse populations.

Most published meta-analyses have predominantly focused on dietary isoflavones and equol in polyphenols and their effects on PCA, while neglecting to explore the relationship between other polyphenol subclasses and polyphenol levels in serum and urine with PCA. Studies such as Yukiko Sugiyama et al. (82) and Catherine C. Applegate et al. (83) may have consequently overestimated their findings and lacked reliability due to this narrow focus. In addition, Jinjing He et al.’s study (84) investigated the relationship between phytoestrogens and PCA risk, but only included isoflavones, genistein, daidzein, and ligans, without further extraction and analysis of the impact of other polyphenol subclasses on PCA risk. Therefore, caution should be exercised when drawing conclusions due to incomplete data extraction and a lack of consistency among the included studies.

Although this meta-analysis provides comprehensive and objective conclusions, it is important to acknowledge several potential limitations. First, variations in study design, study population, sample size, risk assessments, and adjustments for related confounding factors among the included studies may introduce bias and reduce the confidence of the conclusions. To address this, a random-effects model was utilized to evaluate the effect of polyphenols on PCA. Additionally, significant heterogeneity exists in the lifestyle habits and geographical locations of the study population. To mitigate this, relevant data were selected for statistical analysis, and adjustments were made for a maximum number of potential confounding factors to enhance the accuracy of the conclusions. Second, although most studies utilized questionnaires and blood samples to assess polyphenol intake, accurately measuring the specific intake of compounds remains challenging, and deviations in intake assessment may have occurred during data collection. Third, not all trials provided subgroup data, such as data on PCA type and intake subgroups, making it difficult to conduct certain subgroup analyses. Therefore, large-scale observational studies are needed to further validate the relevant conclusions.

Despite its limitations, this meta-analysis possesses several strengths. Firstly, to our knowledge, this study is the first meta-analysis to categorize extracted data by different forms of polyphenols or polyphenol subclasses (dietary, blood, and urine) and to perform subgroup analyses to comprehensively explore the potential effect of total polyphenols and their subclasses on different types of PCA. Secondly, this study included a large number of observational studies, encompassing over 800,000 participants across Europe, Asia, and America. The large observational population increases the reliability and validity of the conclusions of this study. In summary, this meta-analysis provides meaningful insights that may offer a new reference for PCA prevention in the male population.

From the perspective of clinical dietary recommendations, this study suggests that for healthy men, clinicians or nutritionists might consider appropriately reducing the recommendation of foods rich in polyphenols, especially those high in flavanols, flavones, and anthocyanins, such as onions, berries, and kale. For patients with early-stage or localized prostate cancer, this study suggests that clinicians or nutritionists might recommend foods rich in isoflavones, such as soybeans, chickpeas, and kudzu root. However, current research does not provide specific recommended intake levels for different populations. Future clinical studies with larger sample sizes and detailed data are needed to further explore the relationship between polyphenols and prostate cancer.

## 5 Conclusion

This study identified that men who consumed dietary polyphenols had a significantly higher risk of PCA compared to those who rarely or never consumed them, particularly with respect to dietary flavonols, flavanols, and anthocyanins. However, neither total dietary polyphenols nor their subclasses showed an effect on non-localized or high-grade PCA, while dietary isoflavones appeared to reduce the incidence of localized or low-grade PCA. Regarding serum/plasma polyphenols, total polyphenols, genistein, and enterolactone demonstrated potential in reducing the incidence of PCA. Conversely, no association was observed between total or subclass urinary polyphenols and PCA risk. Considering these limitations, further large-scale prospective cohort studies are warranted to validate these findings.

## Data availability statement

The original contributions presented in the study are included in the article/Supplementary material, further inquiries can be directed to the corresponding author.

## Author contributions

YH: Data curation, Writing – original draft, Writing – review & editing. WW: Data curation, Investigation, Methodology, Resources, Writing – review & editing. JJ: Software, Validation, Writing – review & editing.
