# Supplementary material for: Association between polyphenol subclasses and prostate cancer: a systematic review and meta-analysis of observational studies
Source: Front Nutr. 2024 Jul 31;11:1428911. doi: 10.3389/fnut.2024.1428911 (PMC11322767; doi:10.3389/fnut.2024.1428911)
Supplement: Supplementary file 1 [file Table_1.DOCX]

**Supplementary Table 1**. **Characteristics of included observational studies in the meta-analysis**

| Author, year | Study type | Polyphenol subclasses | Exposure assessment | Reference value (control group) | Adjusting parameters |
| --- | --- | --- | --- | --- | --- |
| Knekt P, 2002 | cohort study | flavonoids | questionnaire | flavonoids: <4.3 g/day | sex, age, geographic area, occupation, smoking, body mass index |
| Greenlee H, 2004 | cohort study | isoflavones | questionnaire | isoflavones: 0 g/day | sex, race, education |
| Kurahashi N, 2007 | cohort study | genistein, daidzein | questionnaire | genistein: <13.2 mg/day  daidzein: <8.5 mg/day | age, area, smoking status, drinking frequency, marital status, body mass index, intake of total fatty acids, dairy, vegetables, fruits |
| Mursu J, 2008 | cohort study | flavonols, flavones, flavanones, flavanols, anthocyanidins, flavonoids | questionnaire | flavonols: 0 g/day  flavones: 0 g/day  flavanones: 0 g/day  flavanols: 0 g/day  anthocyanidins: 0 g/day  flavonoids: 0 g/day | age, examination years, body mass index, smoking status, pack-years of smoking, physical activity, intakes of alcohol, total fat, saturated fat, energy adjusted intake of fiber, vitamin C, vitamin E |
| Park SY, 2008 | cohort study | genistein, daidzein, glycitein, isoflavones | questionnaire | genistein: <0.7 mg/1,000 kcal  daidzein: <0.7 mg/1,000 kcal  glycitein: <0.18 mg/1,000 kcal  isoflavones: <0.7 mg/1,000 kcal | time since cohort entry, ethnicity, family history of prostate cancer, education, body mass index, smoking status and energy intake |
| Geybels MS, 2013 | cohort study | flavonols | questionnaire | flavonols: 0 g/day | age |
| Wang Y, 2014 | cohort study | flavonoids, anthocyanidins, flavanols, flavanones, flavones, flavonols, isoflavones | questionnaire | flavonoids: <126.6 mg/day  anthocyanidins: <5.9 mg/day  flavanols: <10.4 mg/day  flavanones: <7.5 mg/day  flavones: <0.5 mg/day  flavonols: <8.9 mg/day  isoflavones: <0.029 mg/day | age, race, family history of prostate cancer, body mass index in 1999, smoking status, aspirin use, total energy intake, history of prostate-specific antigen screening, history of diabetes. |
| Sawada N, 2017 | cohort study | genistein, daidzein | questionnaire | genistein: 0 g/day  daidzein: 0 g/day | NA |
| Reger MK, 2018 | cohort study | isoflavones, genistein, daidzein, glycitein, formononetin, biochanin a, coumestrol | questionnaire | isoflavones: <0.17 mg/day  genistein: <0.04 mg/day  daidzein: <0.11 mg/day  glycitein: <0.001 mg/day  formononetin: <0.005 mg/day  biochanin a: <0.028 mg/day  coumestrol: <0.03 mg/day | age, race/ethnicity, body mass index, smoking status, alcohol intake, family history of prostate cancer. |
| Sawada N, 2022 | cohort study | isoflavones | questionnaire | isoflavones: <13.5 mg/day | age, area, smoking, alcohol frequency, body mass index, leisure time activity, history of diabetes, screening, intake of green tea, coffee, vegetables and fruit |
| Almanza-Aguilera E, 2023 | cohort study | polyphenols, flavonoids, flavanols, flavanols, flavonols, flavanones, anthocyanins, flavones, isoflavones, lignan | questionnaire | polyphenols: <783 mg/day  flavonoids: 0 mg/day  flavanols: 0 mg/day  flavanols: 0 mg/day  flavonols: 0 mg/day  flavanones: 0 mg/day  anthocyanins: 0 mg/day  flavones: 0 mg/day  isoflavones: 0 mg/day  lignan: 0 mg/day | smoking status, physical activity, educational level, marital status, and diabetes prevalence, and alcohol, body mass index, total energy, fiber, vitamin C intakes |
| Strom SS, 1999 | case-control study | isoflavones, genistein, daidzein, formononetin, biochanin a, coumestrol, phytosterols | questionnaire | isoflavones: 0 mg/day  genistein: 0 mg/day  daidzein: 0 mg/day  formononetin: 0 mg/day  biochanin a: 0 mg/day  coumestrol: 0 mg/day  phytosterols: 0 mg/day | age, family history of prostate cancer, alcohol intake, total calorie intake |
| Kolonel LN, 2000 | case-control study | isoflavones | questionnaire, interview | isoflavones: 0 g/day | age, education, ethnicity, geographic area, calories |
| Stattin P, 2002 | case-control study | enterolactone | blood sample | enterolactone: <4.32 nmol/l | NA |
| Lee MM, 2003 | case-control study | genistein, daidzein | interview | genistein: <17.9 mg/day  daidzein: <10.0 mg/day | total calories, age |
| Ozasa K, 2004 | case-control study | genistein, daidzein, equol | questionnaire, blood sample | genistein: <239 nmol/l  daidzein: <89 nmol/l  equol: <1.91 nmol/l | age |
| McCann SE, 2005 | case-control study | lignans | questionnaire | lignans: ≤2 g/day | age, education, body mass index, cigarette smoking status, total energy |
| Hedelin M, 2006 | case-control study | isoflavones, genistein, daidzein, lignans | questionnaire, blood sample | isoflavones: <1.0 g/day  genistein: <0.27 g/day  daidzein: <0.49 g/day  lignans: <17.9 g/day | age, intake of antibiotics, zinc, animal fat, total energy intake, alcohol, vegetable, fat, carbohydrates, red meat |
| Low YL, 2003 | case-control study | daidzein, genistein, glycitein, equol, enterodiol, enterolactone | questionnaire, blood sample | daidzein: 0 μg/mmol creatinine  genistein: 0 μg/mmol creatinine  glycitein: 0 μg/mmol creatinine  equol: 0 μg/mmol creatinine  enterodiol: 0 μg/mmol creatinine  enterolactone: 0 μg/mmol creatinine | family history of prostate cancer, weight, height, and energy intake |
| Heald CL, 2007 | case-control study | isoflavones, daidzein, genistein, equol, enterolactone | questionnaire, blood sample | isoflavones: <58.1 mg/day  equol: 0 nmol/l  daidzein: <8.26 nmol/l  genistein: <14.23 nmol/l  enterolactone: <8.14 nmol/l | age, total energy intake, family history of prostate cancer and breast cancer, Carstairs Deprivation Index, smoking, energy intake |
| Bosetti C, 2009 | case-control study | flavanones, flavanols, flavonols, flavones, anthocyanidins, isoflavones, flavonoids | questionnaire | flavanones: <5.2 mg/day  flavanols: <29.9 mg/day  flavonols: <15.1 mg/day  flavones: <0.2 mg/day  anthocyanidins: <8.3 mg/day  isoflavones: <14.7 mg/day  flavonoids: <109.4 mg/day | age, study center, education, body mass index, family history of prostate cancer, total calorie intake |
| Nagata Y, 2007 | case-control study | isoflavones, daidzein, genistein | questionnaire | isoflavones: <30.5 mg/day  daidzein: <1.1 mg/day  genistein: <0.8 mg/day | cigarette smoking, energy, fatty acids intakes |
| Ward H, 2008 | case-control study | lignans, isoflavones, genistein, daidzein, equol, glycitein, enterodiol, enterolactone | questionnaire, blood sample | lignans: 0 ng/ml  isoflavones: 0 ng/ml  genistein: 0 ng/ml  daidzein: 0 ng/ml  equol: 0 ng/ml  glycitein: 0 ng/ml  enterodiol: 0 ng/ml  enterolactone: 0 ng/ml | age, height, weight, intake of energy, fat, lycopene, whether sample had been analyzed in a prior publication |
| Kurahashi N, 2008 | case-control study | genistein, daidzein, glycitein, equol | questionnaire, blood sample | genistein: <57 ng/ml  daidzein: <22 ng/ml  glycitein: <1.0 ng/ml  equol: <1.0 ng/ml | smoking status, alcohol intake, marital status, intake of green tea, protein, fiber, green or yellow vegetables |
| Lewis JE, 2009 | case-control study | genistein, daidzein | questionnaire | genistein: ≤196 μg/day  daidzein: ≤77 μg/day | age, education, body mass index, smoking history, family history of prostate cancer in first-degree relatives, total caloric intake |
| Travis RC, 2009 | case-control study | genistein, daidzein, equol, lignans, enterolactone, enterodiol | blood sample | genistein: <0.30 ng/ml  daidzein: <0.30 ng/ml  equol: <0.05 ng/ml  lignans: 0 ng/ml  enterolactone: <1.40 ng/ml  enterodiol: <0.10 ng/ml | smoking, physical activity, alcohol intake,marital status, education, body mass index |
| Park SY, 2009 | case-control study | daidzein, genistein, equol, enterolactone | urine sample | daidzein: <0.053 nmol/mg  genistein: <0.009 nmol/mg  equol: <0.0001 nmol/mg  enterolactone: <0.227 nmol/mg | age at specimen collection, fasting hours, family history of prostate cancer, body mass index, education |
| Ward HA, 2010 | case-control study | phytoestrogens, isoflavones, genistein, daidzein, glycitein, biochanin a, formononetin, lignans, enterolactone, equol, coumestrol | questionnaire, blood sample | phytoestrogens: 0 μg/mmol creatinin  isoflavones: 0 μg/mmol creatinin  genistein: 0 μg/mmol creatinin  daidzein: 0 μg/mmol creatinin  glycitein: 0 μg/mmol creatinin  biochanin a: 0 μg/mmol creatinin  formononetin: 0 μg/mmol creatinin  lignans: 0 μg/mmol creatinin  enterolactone: 0 μg/mmol creatinin  equol: 0 μg/mmol creatinin  coumestrol: 0 μg/mmol creatinin | age, height, weight, physical activity, social class, family history of prostate cancer, daily intake of energy, fat, zinc, selenium, lycopene, total intake of dairy products |
| Jackson MD, 2010 | case-control study | genistein, daidzein, equol, enterolactone | questionnaire, blood sample, urine sample | genistein: <0.155 nmol/mg creatinine  daidzein: <0.117 nmol/mg creatinine  equol: <0.035 nmol/mg creatinine  enterolactone: <0.550 nmol/mg creatinine | age, alcohol, body mass index, education, family history of prostate cancer, physical activity, antibiotic use, smoking |
| Sawada N, 2010 | case-control study | genistein, equal | questionnaire, blood sample | genistein: <86.2 ng/ml  equal: <1.0 ng/ml | testosterone, sex hormone-binding globulin, smoking status, alcohol intake, marital status, body mass index, intake of green tea and miso soup |
| Travis RC, 2012 | case-control study | genistein | blood sample | genistein: <0.30 ng/ml | smoking, physical activity, alcohol intake, marital status, education, body mass index |
| Sugiyama Y, 2014 | case-control study | genistein, daidzein, glycitein, equol | blood sample | genistein: ≤59.3 ng/ml  daidzein: ≤19.3 ng/ml  glycitein: ≤1.0 ng/ml  equol: <0.5 ng/ml | age |
| Wu Y, 2015 | case-control study | genistein | blood sample, interview | genistein: <640.2 nmol/l | age |
| Nagata Y, 2016 | case-control study | genistein, daidzein, glycitein, equol | questionnaire, blood sample, fecal sample | genistein: <57.10 ng/ml  daidzein: <18.00 ng/ml  glycitein: <0.80 ng/ml  equol: <0.50 ng/ml | age, body mass index, total energy intake, smoking, alcohol status |
| Russo GI, 2017 | case-control study | lignans, isoflavones, daidzein, genistein, glycitein, biochanin a | questionnaires | lignans: 0 g/day  isoflavones: 0 g/day  daidzein: 0 g/day  genistein: 0 g/day  glycitein: 0 g/day  biochanin a: 0 g/day | age, energy intake, weight status, smoking status,  alcohol consumption, physical activity level, family history of prostate cancer |
| Reale G, 2018 | case-control study | flavonoids, anthocyanins, flavonols, flavanols, flavanones, flavones | questionnaire | flavonoids: 0 g/day  anthocyanins: 0 g/day  flavonols: 0 g/day  flavanols: 0 g/day  flavanones: 0 g/day  flavones: 0 g/day | age, energy intake, weight status, smoking status, alcohol consumption, physical activity level, family history of prostate cancer |
| Ghanavati M, 2021 | case-control study | flavonoids, lignans, polyphenols, anthocyanins, flavonols, flavanols, flavanones, flavones | questionnaire | flavonoids: <718.29 mg/day  lignans: <8.76 mg/day  polyphenols: <2287.19 mg/day  anthocyanins: <23.17 mg/day  flavonols: <438.74 mg/day  flavanols: <94.27 mg/day  flavanones: <58.33 mg/day  flavones: <3.35 mg/day | energy intake, hypertension, diabetes, smoking, body mass index and waist circumstance |
| Galván-Portillo M, 2021 | case-control study | flavones, flavonols, flavanols | questionnaire, interview | flavones: 1.0 mg/day  flavonols: 1.0 mg/day  flavanols: 1.0 mg/day | age, educational level, history of chronic disease, history of sexually transmitted disease, history of prostate cancer in first-degree relatives, leisure physical activity and smoking patterns throughout life, raw tomato, green-yellow leafy vegetables, green-yellow nonleafy vegetables |
