# Supplementary material for: Association between polyphenol subclasses and prostate cancer: a systematic review and meta-analysis of observational studies
Source: Front Nutr. 2024 Jul 31;11:1428911. doi: 10.3389/fnut.2024.1428911 (PMC11322767; doi:10.3389/fnut.2024.1428911)
Supplement: Supplementary file 2 [file Table_2.DOCX]

**Supplementary Table 2.** **Quality assessment of cohort studies included.**

| Author, year,  Study (Observational) | Selection (Out of 4) | | | | Comparability  (Out of 2) | Outcomes (Out of 3) | | | Total  (Out of 9) |
| --- | --- | --- | --- | --- | --- | --- | --- | --- | --- |
|  | Representativeness of exposed cohort | Selection of non exposed cohort | Ascertainment  of exposure | Outcome not present at the start of the study |  | Assessment of outcomes | Length of follow-up | Adequacy of follow up of cohorts |  |
| Knekt P, 2002 | 1 | 1 | 1 | 1 | 2 | 0 | 1 | 1 | 8 |
| Greenlee H, 2004 | 1 | 0 | 1 | 1 | 1 | 1 | 1 | 1 | 7 |
| Kurahashi N, 2007 | 0 | 1 | 1 | 1 | 2 | 1 | 1 | 1 | 8 |
| Mursu J, 2008 | 0 | 1 | 0 | 1 | 1 | 1 | 1 | 1 | 6 |
| Park SY, 2008 | 1 | 1 | 1 | 1 | 2 | 1 | 1 | 0 | 8 |
| Geybels MS, 2013 | 1 | 1 | 1 | 0 | 2 | 0 | 1 | 1 | 7 |
| Wang Y, 2014 | 1 | 1 | 1 | 1 | 0 | 1 | 1 | 1 | 7 |
| Sawada N, 2017 | 0 | 1 | 1 | 1 | 2 | 1 | 1 | 1 | 8 |
| Reger MK, 2018 | 1 | 1 | 0 | 1 | 1 | 1 | 1 | 1 | 7 |
| Sawada N, 2022 | 1 | 1 | 1 | 1 | 2 | 1 | 1 | 1 | 9 |
| Almanza-Aguilera E, 2023 | 1 | 1 | 1 | 1 | 2 | 1 | 1 | 1 | 9 |

The observational studies were assessed by the Newcastle-Ottawa Quality Assessment Scale (NOS) checklist of cohort studies.
