# Supplementary material for: Association between polyphenol subclasses and prostate cancer: a systematic review and meta-analysis of observational studies
Source: Front Nutr. 2024 Jul 31;11:1428911. doi: 10.3389/fnut.2024.1428911 (PMC11322767; doi:10.3389/fnut.2024.1428911)
Supplement: Supplementary file 3 [file Table_3.DOCX]

**Supplementary Table 3. Quality assessment of case control studies included.**

| Author, year,  Study (Observational) | **Selection (Out of 4)** | | | | **Comparability**  **(Out of 2)** | **Outcomes (Out of 3)** | | | **Total**  **(Out of 9)** |
| --- | --- | --- | --- | --- | --- | --- | --- | --- | --- |
|  | Adequate case definition | Representativeness of the cases | Selection of controls | Definition of controls |  | Ascertainment of exposure | Same method of ascertainment for cases and controls | Non-response rate |  |
| Strom SS, 1999 | 0 | 1 | 1 | 1 | 2 | 1 | 1 | 1 | 8 |
| Kolonel LN, 2000 | 1 | 1 | 1 | 1 | 1 | 1 | 0 | 1 | 7 |
| Stattin P, 2002 | 1 | 0 | 1 | 0 | 2 | 0 | 1 | 1 | 6 |
| Lee MM, 2003 | 1 | 1 | 1 | 0 | 1 | 1 | 1 | 1 | 7 |
| Ozasa K, 2004 | 1 | 1 | 0 | 1 | 2 | 1 | 1 | 1 | 8 |
| McCann SE, 2005 | 0 | 1 | 1 | 1 | 2 | 1 | 1 | 1 | 8 |
| Hedelin M, 2006 | 1 | 1 | 1 | 1 | 1 | 1 | 1 | 1 | 8 |
| Low YL, 2003 | 1 | 1 | 1 | 0 | 2 | 1 | 1 | 1 | 8 |
| Heald CL, 2007 | 1 | 1 | 1 | 1 | 2 | 1 | 1 | 1 | 9 |
| Bosetti C, 2009 | 1 | 1 | 1 | 1 | 2 | 1 | 1 | 1 | 9 |
| Nagata Y, 2007 | 0 | 1 | 1 | 1 | 2 | 1 | 1 | 1 | 8 |
| Ward H, 2008 | 1 | 1 | 1 | 1 | 1 | 1 | 1 | 1 | 8 |
| Kurahashi N, 2008 | 1 | 1 | 1 | 1 | 2 | 1 | 1 | 1 | 9 |
| Lewis JE, 2009 | 1 | 1 | 1 | 1 | 1 | 1 | 0 | 1 | 7 |
| Travis RC, 2009 | 1 | 0 | 1 | 0 | 2 | 0 | 1 | 1 | 6 |
| Park SY, 2009 | 1 | 1 | 1 | 0 | 1 | 1 | 1 | 1 | 7 |
| Ward HA, 2010 | 1 | 1 | 0 | 1 | 2 | 1 | 1 | 1 | 8 |
| Jackson MD, 2010 | 0 | 1 | 1 | 1 | 2 | 1 | 1 | 1 | 8 |
| Sawada N, 2010 | 1 | 1 | 1 | 1 | 1 | 1 | 1 | 1 | 8 |
| Travis RC, 2012 | 1 | 1 | 1 | 0 | 2 | 0 | 1 | 1 | 7 |
| Sugiyama Y, 2014 | 1 | 1 | 1 | 1 | 1 | 1 | 1 | 0 | 7 |
| Wu Y, 2015 | 1 | 1 | 0 | 1 | 2 | 1 | 1 | 1 | 8 |
| Nagata Y, 2016 | 1 | 1 | 1 | 1 | 2 | 1 | 1 | 1 | 9 |
| Russo GI, 2017 | 1 | 1 | 1 | 1 | 2 | 1 | 1 | 1 | 9 |
| Reale G, 2018 | 1 | 1 | 1 | 1 | 2 | 1 | 1 | 1 | 9 |
| Ghanavati M, 2021 | 0 | 1 | 1 | 1 | 2 | 1 | 1 | 1 | 8 |
| Galván-Portillo M, 2021 | 1 | 1 | 1 | 1 | 2 | 1 | 1 | 1 | 9 |

The observational studies were assessed by the Newcastle-Ottawa Quality Assessment Scale (NOS) checklist of case control studies.
